# How could we enhance translation of sepsis immunology to inform immunomodulation trials in sepsis?

**DOI:** 10.1186/s13054-017-1715-0

**Published:** 2017-05-26

**Authors:** M. Shankar-Hari

**Affiliations:** 1 Guy’s and St Thomas’ NHS Foundation Trust, London, SE17EH UK; 2 Division of Immunology, Infection & Inflammatory Disease, Kings College London, London, SE1 9RT UK; 3 Intensive Care National Audit & Research Centre, Napier House, 24 High Holborn, London, WC1V 6AZ UK; 4 ICU Secretaries Offices, St Thomas’ Hospital, Guy’s and St Thomas’ NHS Foundation Trust, London, SE17EH UK

**Keywords:** Sepsis, Immunology, Trials, Host response

## Abstract

Sepsis results in complex alterations to the immune system. Our understanding of how these alterations in immune responses could help characterize extreme immune phenotypes, identify biomarkers with the ability to stratify patients for therapeutic interventions, surrogates in the causal pathway of clinical end-points, and treatable traits are still rudimentary. A methodologically rigorous, consensus-based approach should enrich sepsis immune subpopulations to increase the probability of successful trials.

## Main Text

Sepsis represents life-threatening organ dysfunction caused by a dysregulated host response to infection [1], which potentially affects every organ system. Susceptibility to damage, repair, and residual sequelae varies markedly between both individuals and organs [2], as do the risk for and outcomes from sepsis, which represent heterogeneity [3]. Studying the temporal effects of sepsis on the immune system is challenging as numerous abnormalities differ between sepsis patients and within the same patient over time [4]. Furthermore, the time between onset of infection to clinical presentation varies considerably, influenced by patient characteristics, infection site, pathogen virulence, and access to healthcare. While novel interventions are frequently discovered and tested, numerous trials are statistically negative [3]. While these interventions may indeed be completely ineffective, it is perhaps more plausible that a benefitting subset is diluted by the overall lack of signal or even harm [5]. Thus, reassessing our specialty’s approach to targeting the dysregulated immune system in sepsis is key.

Recently, Antonakos et al. [6] replicated the often reported finding that persistent impaired ex vivo cytokine production of monocytes and lymphocytes stimulated with either lipopolysaccharide (LPS) or Pam3 seen in sepsis patients differs by survival status [4, 7]. LPS is a conserved motif on Gram-negative bacteria. Pam3 is a Toll-like receptor agonist.

The *causal* reasoning here and in similar studies is that impaired cytokine production is a therapeutically modifiable surrogate endpoint that can improve outcomes in sepsis. This reasoning has not helped so far in bringing new therapies to routine clinical use [5]. In this editorial, I suggest that enhanced translation and smarter interpretation of the sepsis immunology knowledge base should derive *extreme immune phenotypes*, clarify *biomarkers*’ purpose, identify *surrogates* in the causal pathway of *clinical outcomes*, and define *treatable traits* within sepsis cohorts (Table 1).

**Table 1:** Definitions of terminology

Terminology	Definition
Extreme phenotypes	Subpopulations defined by extremes of clinical features and outcomes
Biomarker	Characteristic that is objectively measured and evaluated as an indicator of normal biologic processes, pathogenic processes, or pharmacologic responses to a therapeutic intervention
Clinical outcome	Characteristic that reflects how a patient feels, functions, or survives
Surrogate outcome	Substitute for clinical endpoints (or outcome) and expected to predict clinical benefit or harm based on epidemiologic, therapeutic, pathophysiologic, or other scientific evidence
Precision medicine	Refers to an approach for disease treatment and prevention that considers individual variability in genes, environment, and lifestyle
Heterogeneity	The differences in the risk of developing sepsis, risk of suffering sepsis-related outcomes, and in treatment response
Treatable traits	Selecting a patient population with a well-defined treatment response characteristic

This is an original table produced by the author for the purposes of this article using references [3, 11]

## Extreme immune phenotypes in sepsis

The complex immune system alterations seen in sepsis separate into two patterns, primarily based on mechanisms contributing to late deaths [4, 7, 8]. In both these host response patterns, pro-inflammatory, anti-inflammatory, and immunosuppression responses are activated at onset of sepsis and early deaths occur because of excessive innate immune system-driven inflammation. Recovery in both patterns is characterised by resolution of inflammation and recovery of immune cell paresis. However, late deaths occur either due to progressive immune cell paresis resulting in secondary infections or due to intractable inflammation-induced organ injury or a combination of immunosuppression and persistent inflammation [4, 7, 8]. These patterns imply that there are at least two extreme immune phenotypes within sepsis cohorts. For example, Davenport et al. [9] identified two sepsis immune phenotypes in critically ill adults with sepsis using whole leukocyte transcriptomics. About 40% of patients had an immunosuppressed phenotype with impaired antigen processing ability suggested by endotoxin tolerance and T-cell exhaustion. This subgroup had a significantly higher mortality. However, are we to infer that the remainder of the cohort had no immunomodulation potential? Of note, much higher validation cohort mortality in this study exemplifies outcome heterogeneity in sepsis.

## Biomarkers to stratify patients for interventions and treatable traits

It is imperative to clarify the ability of numerous biomarkers reported in sepsis literature [10] to either diagnose, predict, prognosticate, and/or act as surrogate outcomes [11]. For example, in the trial by Meisel and colleagues [12] using granulocyte-monocyte colony stimulating factor (GM-CSF), HLD-DR is positioned as a diagnostic biomarker for immunosuppression and as a surrogate outcome for intervention, with a tenuous link to reported clinical outcomes. The clinical outcomes that improved were duration of mechanical ventilation and hospital stay [12]. Interestingly 15% of patients in the control arm spontaneously restored their HLA-DR expression, which implies that HLA-DR also identifies placebo responders. Promising interventions in sepsis include interleukin-7, programmed cell death pathway specific antibodies, interferon-γ, and GM-CSF [4]. These therapies will need different biomarkers for stratification, response prediction, and to work as surrogate outcomes. This also highlights the need to match intervention with *treatable traits* to accomplish precision medicine [3].

## Surrogates in the causal pathway of clinical end-points

Causal models represent a directional link between variables and their associated probabilities for a given set of clinical circumstances. Therefore, it is important that when trials report surrogate outcome(s), similar inferences should be possible about likely clinical outcome(s). Let us consider nosocomial infection as an example to discuss this issue. Nosocomial infection is a difficult outcome to define, its risk varies with time, and it competes with mortality for event rate as it is associated with greater illness severity, more inflammation, and greater activation of endothelial markers [13]. The attributable mortality when compared to non-sepsis controls is not significantly higher [14]. Thus, the surrogate outcome should ideally mirror these relationships observed with clinical outcomes and should have a causal link.

In summary, our understanding of intervention-matched extreme immune phenotypes and outcomes in sepsis trials is not sophisticated enough to yield positive results. Whilst a moratorium on trials is unreasonable, a consensus towards study designs using fundamental principles of population epidemiology and biological response characterisation for immunomodulation trials is not.

## Abbreviations

GM-CSF: Granulocyte-monocyte colony stimulating factor
HLA: Human leukocyte antigen
LPS: Lipopolysaccharide
